# Pain Management during Office Hysteroscopy: An Evidence-Based Approach

**DOI:** 10.3390/medicina58081132

**Published:** 2022-08-20

**Authors:** Giovanni Buzzaccarini, Luis Alonso Pacheco, Amerigo Vitagliano, Sergio Haimovich, Vito Chiantera, Péter Török, Salvatore Giovanni Vitale, Antonio Simone Laganà, Jose Carugno

**Affiliations:** 1 Gynecology and Obstetrics Clinic, Department of Women and Children’s Health, University of Padua, 35100 Padua, Italy; 2 Unidad de Endoscopia Ginecológica, Centro Gutenberg, Hospital Xanit Internacional, 29003 Málaga, Spain; 3 Department of Obstetrics and Gynecology, Hospital del Mar, Autonomous University of Barcelona, 08003 Barcelona, Spain; 4 Department of Obstetrics and Gynecology, Hillel Yaffe Medical Center, The Ruth and Bruce Rappaport Faculty of Medicine, Technion-Israel Institute of Technology, Hadera 8100, Israel; 5 Unit of Gynecologic Oncology, ARNAS “Civico—Di Cristina—Benfratelli”, Department of Health Promotion, Mother and Child Care, Internal Medicine and Medical Specialties (PROMISE), University of Palermo, 90127 Palermo, Italy; 6 Department of Obstetrics and Gynecology, Faculty of Medicine, University of Debrecen, H-4032 Debrecen, Hungary; 7 Obstetrics and Gynecology Unit, Department of General Surgery and Medical Surgical Specialties, University of Catania, 95124 Catania, Italy; 8 Minimally Invasive Gynecology Unit, Obstetrics, Gynecology and Reproductive Sciences Department, Miller School of Medicine, University of Miami, Miami, FL 33136, USA

**Keywords:** anxiety, office hysteroscopy, outpatient hysteroscopy, pain, practical guidelines

## Abstract

*Background and Objectives*: Hysteroscopy is a reliable technique which is highly useful for the evaluation and management of intrauterine pathology. Recently, the widespread nature of in-office procedures without the need for anesthesia has been requesting validation of practical approach in order to reduce procedure-related pain. In this regard, we performed a comprehensive review of literature regarding pain management in office hysteroscopic procedures. *Materials and Methods*: MEDLINE, EMBASE, The Cochrane Library (Cochrane Database of Systematic Reviews, Cochrane Central Register of Controlled Trials, Cochrane Methodology Register), Global Health, Health Technology Assessment Database and Web of Science, other research registers (for example Clinical Trials database) were searched. We searched for all original articles regarding pain relief strategy during office hysteroscopy, without date restriction. Results have been collected and recommendations have been summarized according to the Appraisal of Guidelines for Research and Evaluation (AGREE) tool. Moreover, the strength of each recommendation was scored following the Grading of Recommendations Assessment (GRADE) system, in order to present the best available evidence. *Results*: Both pharmacological and non-pharmacological strategies for pain management are feasible and can be applied in office setting for hysteroscopic procedures. The selection of strategy should be modulated according to the characteristics of the patient and difficulty of the procedure. *Conclusions*: Accumulating evidence support the use of pharmacological and other pharmacological-free strategies for reducing pain during office hysteroscopy. Nevertheless, future research priorities should aim to identify the recommended approach (or combined approaches) according to the characteristics of the patient and difficulty of the procedure.

## 1. Introduction

Hysteroscopy is considered the gold standard procedure for the evaluation and management of intrauterine pathologies [1,2]. Over the last decade, the number of hysteroscopic procedures performed in office setting has increased substantially. Initially, the office setting was limited to diagnostic procedures; however, with advances in hysteroscopic technology, including miniaturization of instruments and improved surgical techniques [3,4,5], an important number of surgical procedures are currently being performed in the office rather than in surgical room with anesthesia. Diagnostic hysteroscopy is a procedure with primary purposes related to patients with abnormal uterine bleeding (AUB) [6,7], infertility, intrauterine retained products of conception [8], suspected Müllerian anomalies [9,10], defects of the Cesarean section scar (isthmocele) [11,12], among other common intrauterine abnormalities [13]. Office hysteroscopy is an efficient procedure with a recognized value for providing adequate visualization of the uterine cavity, with great patient acceptability, and a negligible complication rate. For this reason, office hysteroscopy is often preferred instead of hysteroscopy performed in the operating room [14], although it is occasionally reported as a painful procedure [15]. Moreover, patient’s anxiety before and during the procedure is the most common reason of failure to conclude the procedure in the office setting. The anxiety increases the perception of pain and limits the tolerability of the procedure. When performing an in office hysteroscopic procedure on a patient who is reporting anxiety, there are several strategies that have been proposed aiming to decrease the perception of anxiety and pain. Both pharmacological (such as non-steroidal anti-inflammatory drugs, antispasmodics, cyclooxygenase-2 inhibitors, local anesthetics, opioids) and non-pharmacological strategies (transcutaneous electrical nerve stimulation—TENS), use of warm distension medium, hypnosis, music) were found to be effective [16]. Moreover, the size of the instrument is known to be of great importance when dealing with pain. For this reason, instrument size reduction plays a key role both for pain reduction and for reducing the risk of vasovagal reactions [17]. A combined approach may be the most efficient strategy to reduce pain during in office hysteroscopic procedures. This approach could include oral medications, but also a dedicated emotional support by healthcare assistant, and visual or auditory sources of entertainment and distraction [18]. Considering this background, the purpose of this comprehensive review is to propose an updated guideline with practical implications for the office hysteroscopy pain and anxiety relief. In particular, the role of the gynecologist in pain management has been investigated, aiming to determine the best strategy that should be utilized by the gynecologist before, during, and after the office hysteroscopy procedure.

## 2. Materials and Methods

A thorough literature review was performed with a search on the following databases: MEDLINE, EMBASE, The Cochrane Library (Cochrane Database of Systematic Reviews, Cochrane Central Register of Controlled Trials, Cochrane Methodology Register), Global Health, Health Technology Assessment Database and Web of Science, other research registers (for example Clinical Trials database); we used the medical subject heading (MeSH) term: “Hysteroscopy” (MeSH Unique ID: D015907) in combination with “Pain Management” (MeSH Unique ID: D059408) and “Outpatients” (MeSH Unique ID: D010045). We considered as suitable only papers written in English, published from databases inception until 31 January 2022.

Titles and abstracts of the studies retrieved using the above-mentioned search strategy were screened by three review authors. They independently identified studies that could answer the objectives of this review. The full text manuscript of all the potentially eligible articles was retrieved and assessed for eligibility and inclusion in this review by another co-author. A third collaborator was inquired when disagreement occurred between reviewers regarding the possible eligibility of particular articles. Data from the articles were extracted by two authors regarding the study features, the included populations, the type of intervention and any outcomes. Similarly, a third co-author was inquired when some disagreement occurred. Due to the features of our results, we decided for a narrative description of the results from the selected articles. For each subsection, recommendations were provided according to the Appraisal of Guidelines for Research and Evaluation (AGREE) tool [19], and strength of each recommendation was scored according to the level of evidence following to the Grading of Recommendations Assessment (GRADE) system [20].

## 3. Results

### 3.1. The Effect of Anxiety, Duration of the Procedure and Operator Experience on Reported Procedural Pain during in Office Hysteroscopy

Outpatient hysteroscopy is associated with pre-procedural and procedural anxiety. Anxiety, other than hampering the patient quality of life and procedure satisfaction, could be associated to pain perception [21]. Frequently, the anxiety level is detected through the use of the State–Trait Anxiety Inventory (STAI) test. However, new proposals have been developed such as the Surgical Anxiety Questionnaire (SAQ) [22] and the Surgical Fear Questionnaire (SFQ) [23].

Jawaid et al. performed a study to ascertain the preoperative anxiety level in patients admitted for an elective surgical procedure. The most common factors that are related to anxiety were fear of complications, postoperative pain, and results of operation [24]. In addition, in 2004, Gupta et al. performed a study aimed to establish women’s experiences during diagnostic and therapeutic hysteroscopy procedures. The main objective was to quantify the anxiety suffered by the patients and to identify predictors of anxiety, using the STAI test. In office hysteroscopy was associated with greater anxiety, which may affect the probability of tolerance for the procedure. However, among those women undergoing operative hysteroscopies, dissatisfaction was not related with the in-office procedure setting. In particular, women presenting to the office for a hysteroscopic procedure reported significantly higher anxiety than women seen at the same gynecology clinic for other reasons. High levels of anxiety and intra-operative procedural pain, but not the specific operative intervention, were significant predictive factors of patients asking for having a repeated procedure (if needed) to be administrated under general anesthesia [25]. Although the correlation between preprocedural anxiety and pain experienced during hysteroscopy is well established [26], it is important to note that the management of anxiety can strongly decrease the need for analgesia [27]. Other risk factors investigated were nulliparity, menopause, personal history of dysmenorrhea, and anxiety which are considered risk factors for reporting greater pain during gynecologic procedures [18].

In 2014, Kokanali et al. designed an observational study including 148 patients aiming to define the correlation between anxiety levels screened before procedure and procedure-related pain in women undergoing office hysteroscopy. In this study, the STAI test was used for anxiety detection and the VAS score for pain measurement. Results showed positive correlations between pre-procedural waiting time, pre-procedural trait, or state anxiety scores, and reported pain level during the procedure [28].

An interesting finding of the above-mentioned study was the correlation between in-hospital pre-procedural waiting time, which, other than the hysteroscopy itself, is correlated to anxiety and lack of pain relief. Based on this, in 2012, Carta et al. performed an observational study including 284 patients investigating the link between the waiting time between pre-procedure counseling with the healthcare specialist and the administration of office hysteroscopy, and the perception of pain. The STAI test was also used to detect anxiety, and the VAS score to detect pain. Interestingly, the waiting time was found slightly correlated with the procedural pain, but not with the anxiety state and trait [29]. According to these elements, significant effort must be provided to minimize the waiting time for the patient, since it is a significant factor affecting patients’ anxiety [26,30].

Moreover, Kokanali et al. provided evidence about the correlation between pain and the duration of the procedure [28]. As expected, the authors found that longer procedures were associated with more pain. This result could be considered in line with another study performed in office setting, which highlighted that the hysteroscopist experience was protective against unacceptable pain during office hysteroscopy [31]. In this regard, adequate experience of the operator allows for short procedural time, and this correlate with less perceived pain. From this perspective, we suggest reducing the procedural time as much as possible, and to schedule the patient for office hysteroscopy when the condition to be treated is suitable for the operator experience. Conversely, when clinician’s perspective is directed towards an operative hysteroscopy with anesthesia, the office hysteroscopy should not be considered suitable, even as an attempt, since it can become painful and not resolutive for the patient.

#### Recommendations for the Management of Women Undergoing Office Hysteroscopy

Based on the available evidence, we promote the following recommendations:

- Strategies aimed to reduce the anxiety level are useful to improve comfort and decrease pain during in office hysteroscopy (Level A).
- Waiting time before office hysteroscopy should be minimized as much as possible, since it is associated with increased pain levels (Level B).
- Procedural time should be reduced as much as possible, scheduling the patient for office hysteroscopy when the condition to be treated is suitable for the operator experience (Level A).

### 3.2. Neuronal and Anatomical Features of Pain in Patients with Endometriosis/Adenomyosis

The importance of pain during a gynecological procedure needs to be fully understood in its anatomical background. In 2010, Zhang et al. performed a retrospective study with immunohistochemical staining on women who presented uterine fibroids (*n* = 37) and women with adenomyosis (*n* = 29). Their objective was to assess whether the endometrium and myometrium of women with painful uterine fibroids and adenomyosis present nerve fibers. For this reason, immunohistochemical markers were used such as polyclonal rabbit antiprotein gene product 9.5 (PGP9.5), which is a pan-neuronal biomarker, and antineurofilament protein (NF). Results from the study showed the statistically significative presence of PGP9.5-immunoactive nerve fibers located in the endometrium of women with pain. Differently, they were not present in women without pain, suggesting a role of PGP9.5-immunoreactive nerve fibers in pain generation [32].

This issue stimulated further research: in 2015, Di Spiezio Sardo et al. performed a prospective case-control study including 198 premenopausal women during fertility assessment to evaluate if nerve fibers were present in the endometrium of patients undergoing office hysteroscopy. Moreover, they aimed to assess their potential active role to the pathogenesis of pain in the intraoperative setting [33]. They used the VAS Score and divided the patients in two group: Group A (case) with a VAS score > 5 and Group B (control) with VAS < 5. For nerve fibers detection, they used immunohistochemical analyses for nerve fiber biomarkers (S100, NSE, SP, VIP, NKR1, NKR2, NPY, NKA, NKB, and NKR3) in the endometrial layer. Their findings showed that S-100, NK-B, NSE, NKR1, NK-A, VIP, and NPY were significantly increased (*p* < 0.01) in endometrial samples from patients with VAS score > 5. Interestingly, confirming the findings of the previous study [32], patients of group A presented a statistically significant higher prevalence of endometriosis and/or adenomyosis rather than group B [33].

In 2021, Yadav et al. designed a retrospective cohort study on 247 women with dysmenorrhea or chronic pelvic pain, aiming to determine the presence of nerve fibers in their endometrium and the correlation of them with VAS score and presence of endometriosis. Among them, in 190 patients who were recruited for undergoing hysterectomy or endometrial biopsy, endometriosis was confirmed, while 57 suffered from dysmenorrhea without endometriosis. For nerve fibers detection, the immunohistochemical staining for PGP9.5 was performed, and nerve fiber density was assessed in endometrial tissue biopsies. As consequence, nerve fibers were detected in slices of the endometrium and myometrium in 68% of patients with endometriosis who underwent hysterectomy or a deep endometrial biopsy. On the contrary, nerve fibers were not detected in the aspirated endometrium of women affected by endometriosis. Differently, 13.7% of women with adenomyosis and 3.3% of women presenting fibroids showed the presence of nerve fibers in their endometrial layer. More importantly, nerve fiber density was linked with pain, evaluated with validated scores, in women with endometriosis. Possibly, the presence of nerve fibers in the eutopic endometrial layer could be used as a diagnostic tool for detecting the presence of endometriosis with a specificity of 92.7%. However, it is important to note that the absence of nerve fibers is not correlated with the absence of the disease [34].

Further interests have been directed into genetic polymorphisms investigations. Indeed, in order to find possible correlations among symptoms during the procedure and serotonin concentration, a genetic polymorphisms analysis have been proposed. In particular, polymorphisms of the serotonin receptors have been considered responsible for affecting the intensity of postoperative pain, and the evaluation of serotonin concentration have been found as a predictor for the use of opioid for pain relief [35].

Regarding the possible clinical predictors, personal history of severe dysmenorrhea was identified as one of the main predictors for unacceptable pain during hysteroscopy [31]. However, this correlation is under investigation. Moreover, other predictors related to pain development have been identified in the presence of cervical stenosis and the duration of the procedure; conversely, multiparity have been proposed as a protective factor [36]. Interestingly, the location of the lesion, when present, did not influence the perception of pain during the procedure. For this reason, the location of the lesion should not be considered as a technical limitation [37]. Finally, pain perception has been found to be mostly linked to operator experience [38]. Based on the evidence, we believe that the operator role has two very important aspects: first, the gynecologist is responsible for pain management during and after the procedure; secondly, the operator must use adequate technique and take advantage of miniaturized instruments.

#### Recommendations for Pain Management in Women with Known or Suspected Adenomyosis or Endometriosis Undergoing Outpatient Hysteroscopy

Considering on the available evidence, we provide the following recommendations:

- Hysteroscopists should pay particular attention for pain management in women who experienced dysmenorrhea, since the level of pain could be significantly higher compared to women without dysmenorrhea (Level B).
- Women with known or suspected adenomyosis/endometriosis needs adequate pain management strategies before undergoing outpatient hysteroscopy, since the level of pain could be significantly higher compared to women without it (Level B).

### 3.3. Non-Pharmacological Strategies

Although a pharmacological approach for pain management during office hysteroscopy seem the most efficient strategy [30], there are also other available options. Even if the real effectiveness of these innovative strategies is under debate, a recent systematic review stresses the usefulness for non-pharmacological options [26].

Interesting approaches in reducing patient’s anxiety before hysteroscopy have considered the use of multimedia. In 2020, Akca et al. tried to answer this insight performing a prospective randomized study on 104 patients investigating the efficacy of video-based multimedia information (MMI) on reducing the anxiety levels for women undergoing office hysteroscopy. Patients were allocated in two groups: the MMI group (*n* = 52) and the one which received conventional written information (*n* = 52). In this study, anxiety level measurement included the STAI, which was administered before the MMI, and the STAI-state (STAI-S) administered after MMI. A Likert scale was used to assess woman’s satisfaction, and the VAS score to assess pain during the procedure. Interestingly, the post-information STAI-S score was significantly decreased in the MMI group rather than that of the group who underwent only written information (*p* < 0.001). Moreover, the satisfaction rate of the MMI group was significantly higher rather than the control group (*p* < 0.001). However, the VAS score of intra-procedural pain was found to be similar for both groups [39]. Other authors support the proposal that providing the patient with pre-procedure adequate information is considered a key non-pharmacological strategy to reduce pain during office hysteroscopy [15].

The use of music has also been considered an interesting non-pharmacological strategy for pain relief: in 2013, Angioli et al. investigated the impact of listening to music during office hysteroscopy on the woman’s perceived pain. They performed a prospective randomized trial including 356 patients to detect the effect of music on anxiety and pain perception during office hysteroscopy. Two groups were created: the listening to music during the procedure group (*n* = 176) and the non-listening to music during the procedure (control) group (*n* = 180), both of which were evaluated through the VAS score and the STAI scale. Interestingly, systolic blood pressure and heart rate were significantly decreased in the group which was administered music compared with the other group. More importantly, women listening to some music presented significantly lower anxiety and pain during and after the procedure [40].

Seven years later, Law et al. performed a prospective randomized trial on 107 patients to assess the effect of music administration in decreasing pain during office hysteroscopy. The patients were divided in two groups: group A, the music group (*n* = 54) and group B, the non-listening to music group (*n* = 53). Intriguingly, women in the listening to music group presented a significantly decreased pain during office hysteroscopy recorded through the VAS score (*p* = 0.02) [41]. Moreover, also a recent position statement suggested the potential role of intraprocedural music to reduce anxiety [30].

#### Recommendations about Non-Pharmacological Strategies for Management of Pain in Women Undergoing Office Hysteroscopy—Before the Procedure

Considering the available evidence, we provide the following recommendations:

- The video-based multimedia information (MMI) administration before procedure is more efficient in anxiety reduction compared to traditional written information (Level B).
- Listening to music during the procedure provides pain and anxiety relief (Level B).

### 3.4. Pharmacological Strategies

We found several studies investigating the use of misoprostol for cervical preparation/ripening.

In 2021, Rund et al. aimed to determine the most efficient time for vaginal administration of dinoprostone in relation to performing office hysteroscopic procedures. For this reason, they performed a randomized double-blind controlled trial including 180 nulliparous patients. Participants were divided into two groups: in group A, defined as the long-interval group, 3 mg of dinoprostone was vaginally located 12 h before the office hysteroscopic procedure; in group B, defined as the short-interval group, the same dose of dinoprostone was administered 3 h before the procedure. For measuring pain, the VAS score was intra-procedural administered and after 30 min. Moreover, other outcome measures were the ease of entry of the hysteroscope into the uterine cavity, the women’s satisfaction score after the procedure, and drug side effects. Women included in the group A (dinoprostone administration 12 h before) presented decreased pain scores during the procedure (*p* < 0.001). However, pain scores evaluated 30 min after the procedure were not different in both groups. Moreover, women reported a greater satisfaction (*p* < 0.001) and the hysteroscope insertion through the cervical canal was easier (*p* = 0.003) and faster (*p* < 0.001) in group with a long-interval dinoprostone rather than the short-interval group. The prevalence of side effects was found similar in both groups [42].

Hameed et al. performed a study comparing the efficacy of vaginally administered misoprostol to intracervical normal saline infiltration with the aim to act as a cervical ripening factor. The patients (*n* = 100) were allocated to two groups: group A (*n* = 50) received 400 mcg vaginal misoprostol 4 h prior surgery; group B (*n* = 50) received intracervical normal saline infiltration immediately before cervical dilatation. This saline infiltration consisted of a total of 10 mL of normal saline administered along the cervical circumference divided into about 10 injections avoiding sites located at hours 3 and 9. The basal cervical dilatation was similar in the two groups, but the normal saline infiltration group presented a time to achieve the desired dilatation significantly shorter. Moreover, the women allocated to the misoprostol group had more complications and it was harder to dilate the cervix than in those allocated to the normal saline infiltration group [43].

In 2014, Issat et al. performed a single blind, placebo-controlled clinical trial to determine the efficacy of ketoprofen compared to intravaginal misoprostol in reducing pain during outpatient hysteroscopy. In this study, 150 patients were allocated in three groups: group A received vaginal misoprostol 400 mcg diluted in 100 mL of 5% glucose solution 4 h before procedure; group B received intravenous ketoprofen 50 mg/mL and a placebo intravaginal solution of 100 mL of glucose solution 5%; group C received vaginal placebo tablets diluted in 100 mL 5% intravenous glucose solution. As primary outcome, pain after hysteroscopy (at 5 min) was found significantly decreased in the misoprostol group compared with the other two groups. However, 15 min after the procedure there were no differences among the three groups regarding VAS score. Taking into account these results, misoprostol administration before procedure may be helpful in reducing pain in office hysteroscopy [44].

In 2020, Gokmen et al. performed a prospective randomized study including 90 patients aiming to determine the effect of rectal misoprostol compared to rectal hyoscine-n-butyl bromide in women undergoing office hysteroscopy. In this trial, patients were divided in three groups: group A (*n* = 30) received placebo, group B (*n* = 30) received 200 mg rectal misoprostol 2 h before the procedure, group 3 (*n* = 30) received 20 mg rectal hyoscine-n-butyl bromide two hours pre-procedure. Interestingly, at 1 h after the procedure, analgesics need resulted lower in patients who received hyoscine-n-butyl bromide (13.3%) compared to placebo (43.3%) and misoprostol (36.7%). However, larger trials are necessary to further determine the role of hyoscine-n-butyl bromide in facilitating pre-procedural cervical dilation [45].

In 2020, Haghighi et al. designed a randomized controlled trial with the aim to compare the ability of vaginal isoniazid (isonicotinic acid hydrazide: INH) and vaginally administrated misoprostol with the aim of cervical ripening before the hysteroscopic procedure. In this study, the authors included postmenopausal women or premenopausal who were nulliparous. The 183 patients were distributed in two arms: the INH group (*n* = 67) underwent a 900 mg of vaginal isoniazid administration (three pills) 8 h before hysteroscopic setting; the misoprostol group (*n* = 45) underwent 400 mcg of vaginal misoprostol administration 8 h before the hysteroscopic procedure. Results showed an easier hysteroscopic access to the uterine cavity (*p* = 0.001) in the INH group (95%) when compared with the misoprostol group (50%) without the need of any additional mechanical dilatation [46].

Finally, a recent systematic review and metanalysis ranked the misoprostol plus intracervical block anesthesia as the most efficient pharmacological therapeutic option for pain management during the procedure followed by misoprostol alone [47].

#### Recommendations about Pharmacological Cervical Preparation/Ripening in Women Undergoing Office Hysteroscopy

Considering all the available evidence, we support the following recommendations:

- The ideal time and dose of dinoprostone administration should be 3 mg vaginally 12 h before office hysteroscopy rather than 3 h before the procedure. This timing is more efficient in reducing pain (VAS score), increase the ease of procedure, and patient’s satisfaction (Level A).
- Misoprostol administration before office hysteroscopy is considered efficient in facilitating cervical priming (Level B) and reducing procedural pain (Level A).
- Vaginal isoniazid is more efficient than misoprostol in cervical priming (Level B).

### 3.5. Technical Variables: Temperature and Pressure of the Distension Medium

Among the different strategies to relieve pain during office hysteroscopy, temperature, and pressure of the distension medium, as well as the use of forceps/morcellators, have a key role. Intuitively, the use of bigger scope diameter seems associated with pain severity. For instance, two previous systematic reviews and meta-analyses found an inverse correlation between scope diameter and office hysteroscopy pain reduction [48,49]. Similarly, regarding the solution for intrauterine infusion, various studies (commented below) have been performed.

An updated systematic review and meta-analysis, including five RCTs with 441 patients, found that warm saline infusion was correlated to an intraprocedural significant VAS pain score reduction compared to the control group (*p* = 0.001). Moreover, the post-procedural VAS pain score was significantly reduced among the warm saline group (*p* = 0.005). Interestingly, women found greater satisfaction with warm saline distension medium infusion rather than the room temperature group (*p* < 0.001) [50].

Other than temperature, one additional parameter can be modified by the gynecologist is the uterine cavity filling pressure. In 2014, Saridogan’s group performed a double-blind, randomized controlled trial on 234 patients with the aim to assess the relationship between uterine filling pressure and hysteroscopy visualization and pain. Specifically, they investigated whether adequate visualization could be achieved with a lower uterine infusion pressures and if patient discomfort, assessed through VAS score, could be reduced. The study included three groups using different uterine filling pressure during hysteroscopy: 40 mmHg (*n* = 77), 70 mmHg (*n* = 78) or 100 mmHg (*n* = 79). Regarding the first outcome, adequate visibility was significantly decreased in the 40-mmHg group (87.0%) rather than the 70-mmHg group (94.9%), and in 100-mmHg group (97.5%). Interestingly, the pain score was not significantly different among all the groups [51]. Similarly, in 2015, Haggag et al. presented a new double-blind randomized controlled trial on 240 patients to compare different intraprocedural uterine solution filling pressure with the aim to determine the most effective pressure for adequate visualization while minimizing pain and increasing patient satisfaction. They allocated participants in three group: group A with a solution filling pressure of 30 mmHg; group B with 50 mmHg, and group C with 80 mmHg. The main outcomes were the ideal visualization of the uterine cavity, the capacity to complete the procedures, the intraprocedural VAS score, the VAS scores at the end of the procedure and 30 min after the procedure. Similar to the previous study [51], adequate visualization was significantly lower in group A (88.7%) when compared to group B (97.5%) and C (and 98.7). However, no difference was found when comparing group B and C. Regarding pain perception during the procedure, differently from the previous study, a progressive higher pain score was documented during the procedure from the lower to the higher pressure groups. Similar results were found immediately after and 30 min after completing the procedure [52]. The use of low-pressure distension medium has also been previously supported [30]. Probably, a good compromise between pain and achieving adequate visualization could be using distension pressure of 60 mmHg [53].

In 2021, De Silva et al. performed a systematic review and metanalysis to identify the optimal distension medium typology, the ideal pressure and the temperature that should be administered to minimize pain during outpatient hysteroscopy [54]. First, normal saline and carbon dioxide distension media showed no significant difference in intraprocedural pain. However, the subgroup analysis of top-quality studies presented a significant reduction in the postprocedural pain, reduction of side-effects, and increase of patient satisfaction when using normal saline as the distention media. Moreover, pressures of ≤40 mmHg decreased intraprocedural pain at the expense of lower visibility (81–89% at ≤40 mmHg vs. 95–99% at ≥50 mmHg). Similarly, postprocedural pain was decreased with media administered with lower pressures. On the contrary, warming the normal saline solution did not reduce intraprocedural pain. Other authors, in a recent systematic review, concluded that there is no difference when using warm saline or room temperature infusion [53]. This systematic review added evidence to a previous systematic review and meta-analysis which assessed the effect of distension medium (normal saline vs. carbon dioxide) on pain during office hysteroscopy. Among this point, Cooper et al. concluded that no difference during the procedure was found between carbon dioxide or normal saline for office hysteroscopy pain [55]. On the contrary, Craciunas et al. concluded that carbon dioxide was linked with greater intraprocedural pain, more episodes of shoulder pain and side effects, decreased patient satisfaction, lower visualization, and greater duration of the procedure [56].

#### Recommendations about Temperature and Pressure of the Distension Medium in Women Undergoing Outpatient Hysteroscopy—During the Procedure

- Using warm saline infusion (41 °C) during office hysteroscopy with the purpose of decreasing pain level during the procedure is not effective (Level B).
- The distention media infusion pressure is correlated to the pain perceived during hysteroscopy and should be at the lowest possible for achieving an adequate visualization (Level B).
- The use of normal saline as distention media is better than carbon dioxide in reducing postprocedural pain, with lower side-effects, and increased patient satisfaction (Level B).

### 3.6. The Role of Analgesic Administration

The administration of analgesics could have an important role in reducing pain during hysteroscopic procedures.

Cicinelli et al. reported that removing endometrial polyps larger than 2 cm and a duration of the procedure of more than 15 min are predictors associated with increased pain during hysteroscopic procedures. Women with previous caesarean section, a history of chronic pelvic pain, anxiety and post-menopausal were found to report higher levels of pain during hysteroscopic procedures [17].

Regarding the analgesic administration, Souza et al. studied the difference between diclofenac and a combination of hyoscine and diclofenac to reduce pain during office hysteroscopy. They performed a randomized, double-blind placebo-controlled clinical trial including 217 patients assessing pain perception during and after outpatient diagnostic hysteroscopy without anesthesia. Three groups were created: group A received placebo; group B received diclofenac sodium 50 mg; and group C received diclofenac sodium 50 mg with Hyoscine-N-Butylbromide 10 mg, administered 1 h before the procedure. In this study, the main outcomes were VAS score after the procedure, the Likert acceptance scale, the post-procedural need for other analgesia, the need to remain in the post-operative room, and the presence of vaso-vagal symptoms. All the three groups had similar VAS scores after the procedure and reported similar procedure acceptance scores. Additionally, in a subgroup analysis of patients treated for chronic pelvic pain, prior medication with diclofenac sodium or hyoscine showed decreased pain levels compared to the placebo [57].

In 2014, Teran-Alonso et al. evaluated the administration of analgesic before office hysteroscopy. For this reason, a prospective randomized study on 200 patients was conducted. Two groups were created: group A (*n* = 100) received 1000 mg paracetamol + 600 mg ibuprofen administered one hour before the procedure; group B (*n* = 100) received no medication. Although the group of patients who received analgesic had lower incidence of nausea, vomiting, and hypotension (*p* = 0.013), no differences in pain scores were observed [58].

Hassan and Haggag in 2016 performed a prospective randomized double-bling placebo-controlled trial on 140 patients to assess the effectiveness of tramadol 50 mg in reducing pain associated with office hysteroscopy. Group A received 50 mg one hour before performing outpatient hysteroscopy, and group B received placebo. The median pain score evaluated through VAS score was significantly decreased in the tramadol group during the procedure (*p* = 0.013), immediately after the procedure (*p* < 0.036), and 30 min after (*p* = 0.034). Only two women in the tramadol group reported nausea, but this did not require cancelation of the procedure [59].

In 2019, Mattar et al. presented a systematic review and meta-analysis of randomized controlled trials to evaluate safety and efficacy of tramadol in pain relief during diagnostic office hysteroscopy. The VAS score during the procedure and 30 min after the procedure was considered as the primary outcome. In this case, tramadol was considered as safe, effective, and efficient in reducing pain during and after diagnostic outpatient hysteroscopic procedures [60].

One other possible intervention that the gynecologist can offer to the patient is the use of transcutaneous electrical nerve stimulation (TENS). If used after the procedure, TENS administration has been found effective in reducing pain, compared to placebo [53]. Other authors considered TENS therapy useful in reducing procedural pain [61]. Moreover, some authors considered it the best non-pharmacological treatment [47]. However, well-designed studies are needed before drawing a conclusion.

A study by Keyhan and Munro [62] investigated the local anesthesia administration for women undergoing both office diagnostic hysteroscopy and operative hysteroscopy. In particular, they investigated the pain scores regarding those patients with a history of previous cesarean or vaginal delivery. Interestingly, those patients presented higher pain scores compared to nulliparous women and there was no difference between cesarean or vaginal delivery.

To conclude, we believe that all the analgesic administration presented in the above studies can be offered to the patient during hysteroscopic procedure.

#### Recommendations for Analgesic Administration in Women Undergoing Office Hysteroscopy—During the Procedure

Considering all the available evidence, we provide the following recommendations:

- Diclofenac sodium administration 1 h before office hysteroscopy is not effective in reducing pain during hysteroscopic procedures (Level B).
- Paracetamol and Ibuprofen administration 1 h before office hysteroscopy is not efficient in reducing pain during hysteroscopic procedures (Level B).
- Tramadol administration before hysteroscopy procedure is safe and efficient in reducing pain during and after diagnostic outpatient hysteroscopy (Level A).

## 4. Conclusions

**Figure 1:**
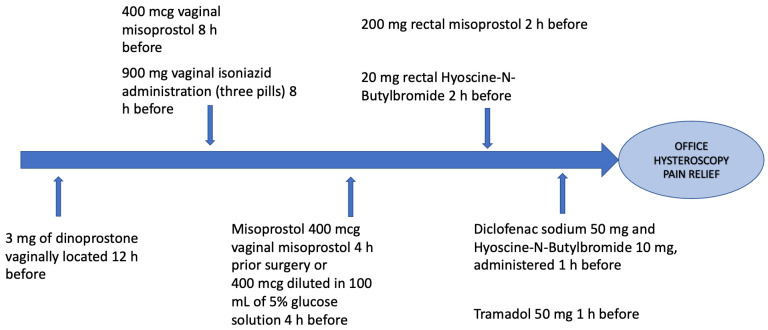
Summary of available pharmacological strategies for pain relief before office hysteroscopy.

During office hysteroscopy, the gynecologist should consider these practical guidelines to minimize pain during hysteroscopic procedures. Pharmacological strategies are summarized in Figure 1 and Table 1; non-pharmacological strategies are summarized in Table 2. In particular, all the possible technical and pharmacological strategies currently available can be efficiently performed by the gynecologist before, during, and after performing office hysteroscopic procedures.

## 5. Stakeholders’ Involvement and Applicability

These recommendations were created based on expert opinion. The main aim was helping the gynecologist treat the average patient during office hysteroscopic procedures. We state that they should not be considered mandatory guidelines and were not built to substitute clinical judgment. Recommendations were decided upon the best available evidence, based on the scientific literature. Moreover, when disagreement was found, the expert panel consensus made a unanimous decision. These guidelines are likely to change as more knowledge of the procedural setting is gained.

## Figures and Tables

**Table 1:** Pharmacological strategies for pain relief in office hysteroscopy setting.

Study	Drgus(s)	Administration
Rund et al. [42]	Dinoprostone	3 mg of dinoprostone vaginally located 12 h before the office hysteroscopic procedure
Hameed and Farhan [43] Haghighi et al. [46] Issat et al. [44] Gokmen Karasu et al. [45]	Misoprostol	400 mcg vaginal misoprostol 4 h prior surgery [43] or 8 h before surgery [46] or 400 mcg diluted in 100 mL of 5% glucose solution 4 h before procedure [44] or 200 mg rectal misoprostol 2 h before the procedure [45]
Gokmen Karasu et al. [45]	Hyoscine-n-butyl bromide	20 mg rectal hyoscine-n-butyl bromide two hours pre-procedure
Haghighi et al. [46]	Isonicotinic acid hydrazide	900 mg of vaginal isoniazid administration (three pills) 8 h before hysteroscopic setting
Souza et al. [57]	Hyoscine and diclofenac	Diclofenac sodium 50 mg and Hyoscine-N-Butylbromide 10 mg, administered 1 h before the procedure
Hassan and Haggag [59]	Tramadol	50 mg one hour before outpatient hysteroscopy

**Table 2:** Non-pharmacological strategies for pain relief in office hysteroscopy setting.

**Study**	**Parameter**	**Strategy**
Carta et al. [29]	Pre-procedural waiting time	Reduction
Kokanali et al. [28]	Intra-procedural waiting time	Reduction
Akca et al. [39]	Video-based multimedia information	Administration before office hysteroscopy
Angioli et al. [40]	Music	Intraprocedural administration
Hameed and Farhan [43]	Intracervical saline infiltration	10 mL of normal saline administered along the cervical circumference divided into about 10 injections avoiding sites located at hours 3 and 9
Shahid et al. [51] Haggag and Hassan [52]	Medium Pressure	The lowest possible for achieving an adequate visualization
Craciunas et al. [56]	Type of medium	Normal saline
Amer-Cuenca et al. [53]	Transcutaneous electrical nerve stimulation (TENS)	After the procedure

## Data Availability

Not applicable.
